# An Analysis of the Gene Expression Associated with Lymph Node Metastasis in Colorectal Cancer

**DOI:** 10.1155/2023/9942663

**Published:** 2023-09-07

**Authors:** Hongjie Yang, Jiafei Liu, Peishi Jiang, Peng Li, Yuanda Zhou, Zhichun Zhang, Qingsheng Zeng, Min Wang, Luciena Xiao Xiao, Xipeng Zhang, Yi Sun, Siwei Zhu

**Affiliations:** ^1^Nankai University, Tianjin, China; ^2^Department of Colorectal Surgery, Tianjin Union Medical Center, Tianjin, China; ^3^Tianjin Institute of Coloproctology, Tianjin, China; ^4^The Institute of Translational Medicine, Tianjin Union Medical Center of Nankai University, Tianjin, China; ^5^Key Laboratory of Molecular Microbiology and Technology, Ministry of Education, TEDA Institute of Biological Sciences and Biotechnology, Nankai University, Tianjin, China; ^6^Department of Mathematics and Statistics, University of Helsinki, Helsinki, Finland; ^7^Department of Oncology, Tianjin Union Medical Center, Tianjin, China

## Abstract

**Objective:**

This study aimed to explore the genes regulating lymph node metastasis in colorectal cancer (CRC) and to clarify their relationship with tumor immune cell infiltration and patient prognoses.

**Methods:**

The data sets of CRC patients were collected through the Cancer Gene Atlas database; the differentially expressed genes (DEGs) associated with CRC lymph node metastasis were screened; a protein–protein interaction (PPI) network was constructed; the top 20 hub genes were selected; the Gene Ontology functions and the Kyoto Encyclopedia of Genes and Genomes pathways were enriched and analyzed. The Least Absolute Shrinkage and Selection Operator (LASSO) regression method was employed to further screen the characteristic genes associated with CRC lymph node metastasis in 20 hub genes, exploring the correlation between the characteristic genes and immune cell infiltration, conducting a univariate COX analysis on the characteristic genes, obtaining survival-related genes, constructing a risk score formula, conducting a Kaplan–Meier analysis based on the risk score formula, and performing a multivariate COX regression analysis on the clinical factors and risk scores.

**Results:**

A total of 62 DEGs associated with CRC lymph node metastasis were obtained. Among the 20 hub genes identified via PPI, only calcium-activated chloride channel regulator 1 (CLCA1) expression was down-regulated in lymph node metastasis, and the rest were up-regulated. A total of nine characteristic genes associated with CRC lymph node metastasis (KIF1A, TMEM59L, CLCA1, COL9A3, GDF5, TUBB2B, STMN2, FOXN1, and SCN5A) were screened using the LASSO regression method. The nine characteristic genes were significantly related to different kinds of immune cell infiltration, from which three survival-related genes (TMEM59L, CLCA1, and TUBB2B) were screened. A multi-factor COX regression showed that the risk scores obtained from TMEM59L, CLCA1, and TUBB2B were independent prognostic factors. Immunohistochemical validation was performed in tissue samples from patients with rectal and colon cancer.

**Conclusion:**

TMEM59L, CLCA1, and TUBB2B were independent prognostic factors associated with lymphatic metastasis of CRC.

## 1. Introduction

Colorectal cancer (CRC) is one of the most common digestive system tumors in the world. It is estimated that there would be 1.93 million new cases of CRC worldwide, accounting for 10.0% of the total cancer incidence and ranking third in the order of cancer incidence [1]. Its incidence rate also suggests an upward trend among patients under the age of 50 years [2]. The postoperative disease-free survival (DFS) of patients under the age of 40 years is significantly lower than that of patients over the age of 40 years [3]. It has been shown that cancer cells of CRC can enter lymphatic vessels, migrate to tumor-draining lymph nodes, grow into lesions in the lymph nodes, and even escape the lymph nodes to spread to other organs [3]. In CRC, the presence of cancer cells in tumor-draining lymph nodes defines stage III disease [4, 5]. For colon cancer, the 5-year survival for patients with stage II (no lymph node metastases) is 79.2–82.5%, in contrast to 59.5–65.4% for patients with stage III disease [6, 7]. The lymph node metastasis of CRC is related to tumor recurrence and overall survival (OS) [8], which is an important marker of tumor progression. Appropriate biomarkers associated with lymph node metastasis may help identify CRC patients at a high risk of recurrence [9]. The incidence of lymph node metastasis in CRC is influenced by many factors, including the stage and location of the tumor, the patient's age, and the presence of lymphatic, venous, and perineural invasion [10, 11]. Whether the tumor features lymph node metastasis connected with the activation of the “metastasis genes” or the inhibition of the “metastasis suppressor genes” of the primary lesion. The signal pathway involved in metastasis is expected to become a therapeutic target for anti-tumor metastasis [12]. In recent years, with molecular tumor targeting and biotherapy as the starting point, it is possible to find target proteins and regulated signal pathways that can be related to the occurrence and development of tumors to achieve better therapeutic effects [13]. Qin et al. [14] discovered that the drug anlotinib can inhibit lymphangiogenesis and lymph node metastasis in patients with lung cancer by inhibiting the phosphorylation of VEGFR-3. Shifting our attention to CRC, Yang et al. [15] identified FSTL3 as a biomarker associated with extracellular matrix (ECM) remodeling and poorer clinical outcomes in CRC. They also suggested FSTL3 as a potential immunotherapeutic target for preventing lymph node metastasis in CRC. Furthermore, Yinhang et al. [16] uncovered an intriguing link between CRC lymph node metastasis and intestinal bacteria, proposing a prediction model based on intestinal bacteria as a new evaluation method. Despite these breakthroughs, the complex mechanisms underlying lymph node metastasis in CRC remain largely unclear. Identifying the genes and signal pathways linked to lymph node metastasis will help provide new therapeutic targets for CRC treatment and provide effective biomarkers for predicting the prognosis of patients. Through a bioinformatics analysis, this study looks for the genes that may lead to lymph node metastasis in CRC patients and discusses the relationship between them and immune cell infiltration, in a bid to provide new ideas regarding the clinical treatment of CRC lymph node metastasis.

## 2. Materials and Methods

### 2.1. Data Sources

The gene expression and clinical data analyzed in this study were sourced from The Cancer Genome Atlas (TCGA, https://portal.gdc.cancer.gov/), which included 476 CRC patients consisting of 281 patients without lymph node metastasis and 195 patients with lymph node metastasis.

### 2.2. Acquisition of Differentially Expressed Genes in Lymph Node Metastasis

The patients were divided into the lymph node metastasis group and the non-lymph node metastasis group. The differentially expressed genes (DEGs) in the lymph node metastasis group and non-lymph node metastasis group were analyzed using the R language limma package [17]. The filter conditions were set as logFCfilter = 1 and fdrFilter = 0.05, and the heatmap and volcano map of the DEGs were plotted.

### 2.3. Protein–Protein Interaction Network Construction and Hub Gene Screening

To define the role of DEGs proteins at the level of the biological network system, the obtained DEGs were imported into the String protein–protein interaction (PPI) database (String, https://cn.string-db.org/) to carry out a PPI analysis, the medium confidence = 0.400 sets to hide the isolated nodes and the protein data exported to build a global network of component target-disease target PPI; PPI data with an interaction score ≥0.40 was taken for network visualization, and the Cytohubba plug-in in the Cytoscape 3.8.0 software was employed to obtain the top 20 genes as hub genes to analyze the expression differences of the hub gene between the lymph node metastasis group and the non-lymph node metastasis group.

### 2.4. Enrichment Analysis

The ClusterProfiler [18] and DOSE [19] package in the R language were utilized to perform Gene Ontology (GO) functions and Kyoto Encyclopedia of Genes and Genomes (KEGG) pathway enrichment analysis on DEGs to obtain the signal pathways related to CRC lymph node metastasis hub genes.

### 2.5. Screening of the Characteristic Genes Associated with Lymph Node Metastasis and the Relationship between Characteristic Genes and Immune Cell Infiltration

Following the approach used by Wu et al. in their research [20], we utilized the Least Absolute Shrinkage and Selection Operator (LASSO), a regression analysis algorithm that leverages regularization to select characteristic genes from the 20 hub genes. The features related to the classification results may be selected, and the R language glmnet package [21] is used for a LASSO regression analysis to further screen the characteristic genes associated with lymph node metastasis from the 20 hub genes. The proportions of each type of immune cell in each sample were estimated using the CIBERSORT algorithm, a robust computational method that employs a deconvolution approach to infer cell type proportions from bulk tissue gene expression profiles. This process involves utilizing the characteristic gene expression levels in each sample and applying a signature matrix of gene expression for 22 immune cell subtypes, as provided by Newman et al. [22]. Following the estimation of immune cell proportions, Spearman's correlation analysis was utilized to determine the relationship between the characteristic genes of lymph node metastasis and immune cell infiltration. The correlation matrix was plotted with the abscissa for genes and the ordinate for immune cells, and the range of the correlation coefficient is set to [−1, 1] with the negative values for negative correlations and the positive values for positive correlations. When *p* < 0.05, the differences are statistically significant.

### 2.6. Construction and Validation of the Prognosis Prediction Model

A uni-factor COX analysis was performed on the characteristic genes of lymph node metastasis. The genes with *p* < 0.05 were used as the survival-related genes; a risk score formula was constructed based on those survival-related genes. The patients were divided into a high-risk group and a low-risk group according to the median risk scores for a Kaplan–Meier analysis. The metric used to assess survival was OS. A Log-Rank test was utilized to compare the differences in the survival curves between the two groups. The SurvivalROC package [23, 24] in the R language was adopted to verify the predictive efficacy of the survival-related genes on OS through the area under the curve (AUC) value of the receiver operating characteristic curve (ROC).

### 2.7. Immunohistochemical Staining

Formalin-fixed, paraffin-embedded (FFPE) sections (5 *μ*m thickness) from rectal cancer and colon cancer were used for immunohistochemical staining experiments. Sections were stained with TMEM59L antibody (ab105417, 2.5 *μ*g/ml, Abcam, Cambridge, UK), calcium-activated chloride channel regulator 1 (CLCA1) antibody (MAB10766, 5 *μ*g/ml, R&D systems, Minneapolis, MN, USA), TUBB2B antibody (sc-47751, 5 *μ*g/ml, Santa Cruz, Dallas, TX, USA). A digital camera was used to capture images of stained sections. The expression of CLCA1, TUBB2B, and TMEM59L in the tissue samples was evaluated and quantified based on the percentage of positive cells. The number of positive cells was visually evaluated as follows: 0 (negative), <5% positive cells; 1 (weak), 6–25% positive cells; 2 (moderate), 26–50% positive cells; 3 (above moderate), 51–75%; and 4 (strong), >76% positive cells [25, 26].

### 2.8. Ethics Statement

Experiments using patient specimens (provided by the Department of Colorectal Surgery, Tianjin Union Medical Center, Nankai University, Tianjin, China), were approved by the Institutional Ethics Committee. Written informed consent was obtained from all patients.

### 2.9. Statistical Analysis

Statistical analysis was performed with the SPSS 20.0 or GraphPad Prism version 8.0. All values were represented as the mean ± SD. The unpaired two-tailed *t*-test was used for the in vitro study and one-way analysis of variance was used for in immunohistochemistry (IHC) study. A two-sided *p* < 0.05 was considered statistically significant (∗*p* < 0.05; N.S. *p* > 0.05).

## 3. Results

### 3.1. Analysis of the DEGs of CRC Lymph Node Metastasis and Non-Lymph Node Metastasis

**Figure 1:**
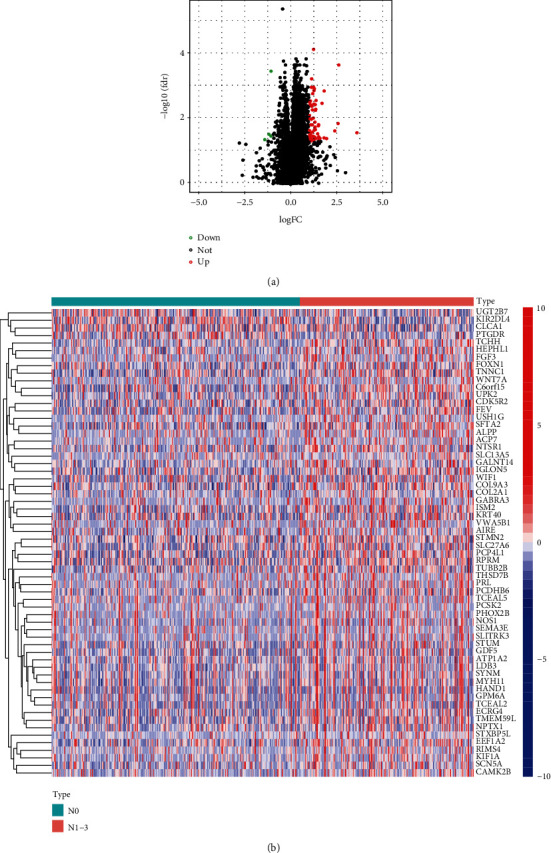
DEGs analysis of whether CRC is involved in lymph node metastasis. (a) Volcano map of mRNA differential expression between CRC lymph node metastasis and non-lymph node metastasis in the TCGA dataset. (b) Heatmap of differentially expressed mRNA in the TCGA dataset (red: up-regulation; blue: down-regulation).

A total of 62 DEGs were obtained between the CRC lymph node metastasis and non-lymph node metastasis, including 4 down-regulated genes (green points) and 58 up-regulated genes (red points; Figure 1(a)). We grouped according to lymph node metastasis (N0 and N1–N3) and performed the heatmap analysis showing different expression genes (Figure 1(b)).

### 3.2. PPI Network Construction

**Figure 2:**
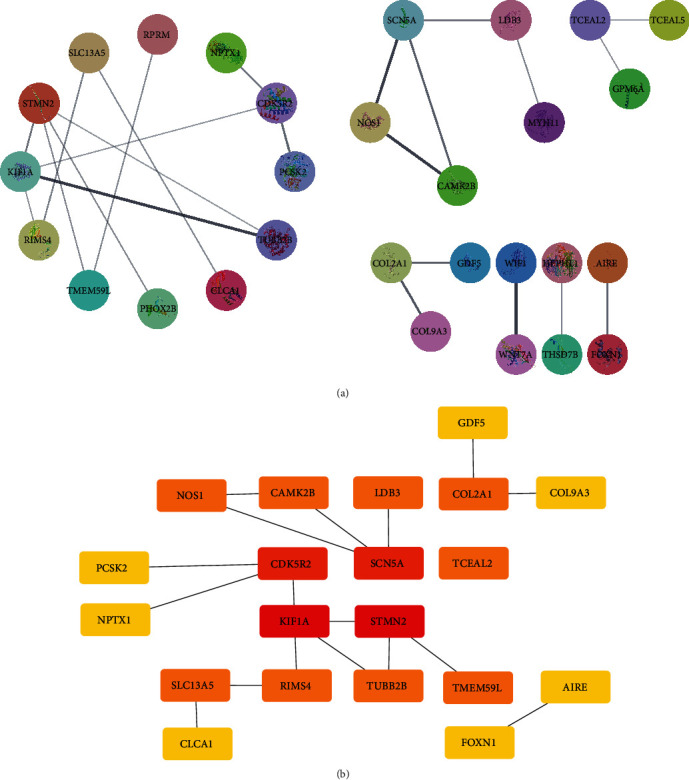
PPI network construction. (a) PPI network of DEGs whether lymph node metastasis occurs. (b) Top 20 hub genes.

A PPI network was constructed based on 4 down-regulated genes and 58 up-regulated genes. The medium confidence = 0.400 was set to hide isolated nodes, and a total of 30 nodes were obtained (Figure 2(a)). The top 20 hub genes were obtained using the Cytohubba plug-in in the Cytoscape 3.8.0 software (Figure 2(b)).

### 3.3. Expression of the Hub Gene in the CRC Tissues with and without Lymph Node Metastasis

**Figure 3:**
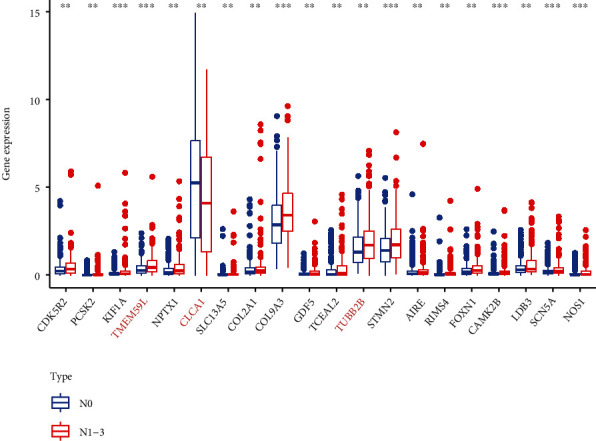
Expression of the 20 hub genes in lymph node metastasis and non-lymph node metastasis (∗*p* < 0.05; ∗∗*p* < 0.01; and ∗∗∗*p* < 0.001).

According to the presence of lymph node metastasis, TCGA samples were divided into a lymph node metastasis group and a non-lymph node metastasis group. The expression of the 20 hub genes between the two groups was observed. The results indicated that among the 20 hub genes, only CLCAI expression was down-regulated in lymph node metastasis, whereas the rest were up-regulated (Figure 3).

### 3.4. GO Analysis and KEGG Analysis of the Hub Genes

**Figure 4:**
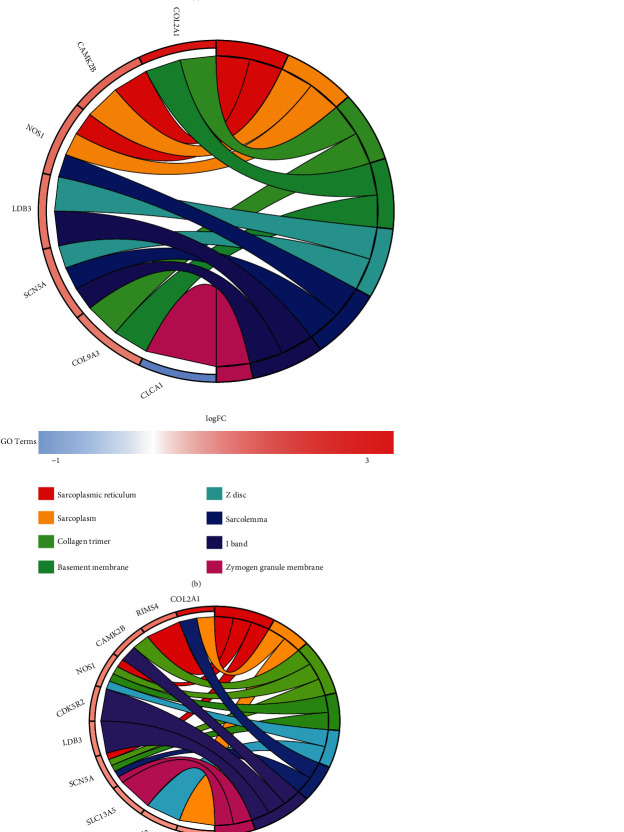
GO analysis and KEGG analysis of the 20 hub genes. (a) Circular diagram of the biological process enrichment analysis. (b) Circular diagram of the cellular component analysis. (c) Circular diagram of the molecular function analysis. (d) Bubble diagram of the KEGG analysis.

GO analysis results (Figures 4(a), 4(b), and 4(c)) showed that these 20 hub genes play an important role in the formation of the sarcoplasmic reticulum, the regulation of postsynapse organization and transmembrane transporter binding. The KEGG analysis results (Figure 4(d)) suggested that these 20 hub genes play important roles in the “Circadian entrainment,” “ECM–receptor interaction,” and “Protein digestion and absorption”.

### 3.5. Screening of the Characteristic Genes of Lymph Node Metastasis and Analysis of the Relationship between the Characteristic Genes and Immune Cell Infiltration

**Figure 5:**
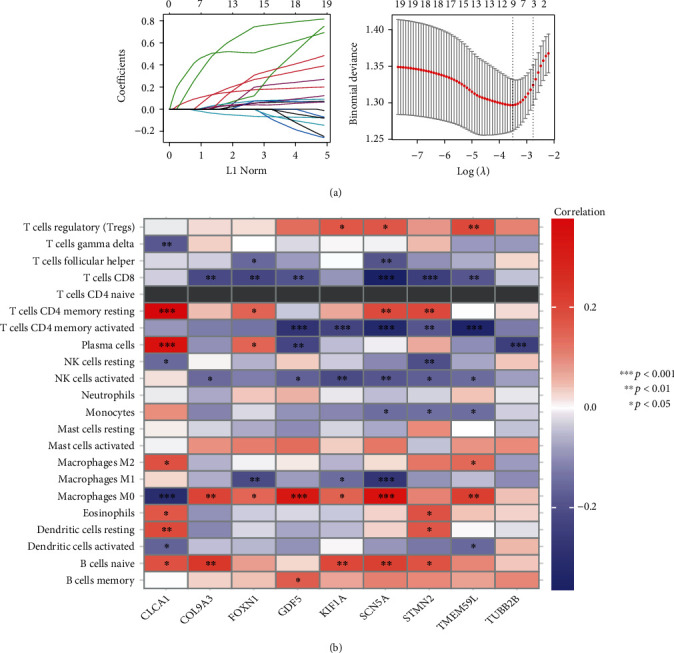
Characteristic gene selection and the relationship between characteristic genes and immune cell infiltration. (a) LASSO screening analysis. (b) Nine characteristic genes and immune cell infiltration level matrix.

A LASSO regression analysis was performed on the 20 hub genes from the CRC patient cohort, and 9 characteristic genes associated with lymph node metastasis were screened, including KIF1A, TMEM59L, CLCA1, COL9A3, GDF5, TUBB2B, STMN2, FOXN1, and SCN5A (Figure 5(a)). The analysis showed that the nine characteristic genes were linked to immune cell infiltration (Figure 5(b)).

### 3.6. Prediction Model

**Figure 6:**
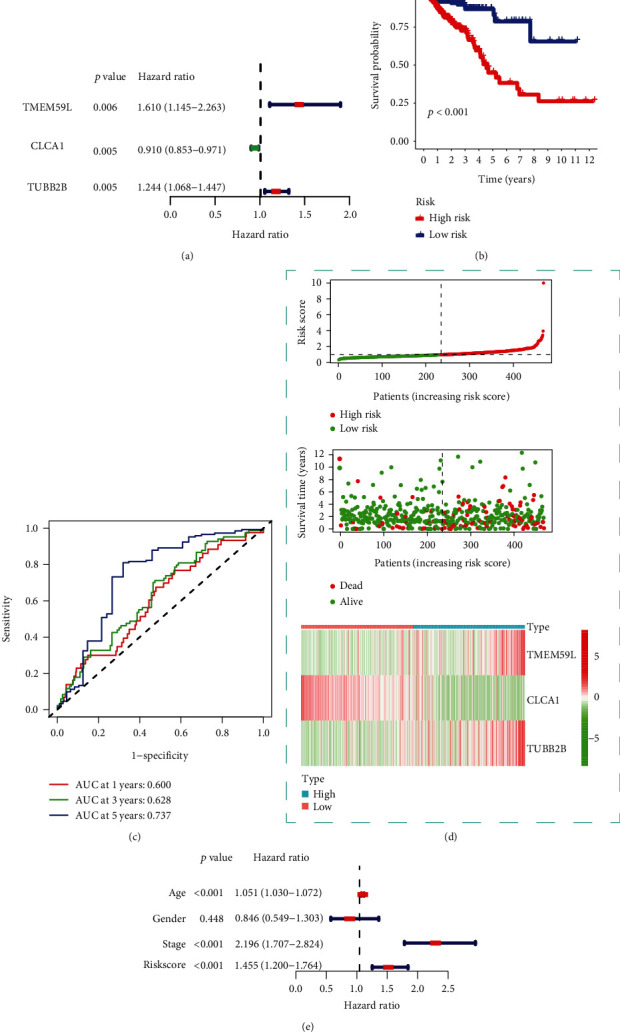
The association between survival-related genes and patient prognosis information. (a) Uni-factor COX analysis results. (b) Kaplan–Meier survival analysis results. (c) Model ROC curve. (d) Risk score, survival time, and survival status in the TCGA dataset. Top: scatterplot of the risk scores from low to high; middle: scatterplot of the survival time and survival status corresponding to the risk scores of different samples. Bottom: heat map of the gene expression in the prognostic model. (e) Multi-factor COX analysis results.

A uni-factor COX analysis was performed on the characteristic genes of lymph node metastasis, and three survival-related genes (*p* < 0.05) were obtained, including TMEM59L, CLCA1, and TUBB2B (Figure 6(a)). The risk score formula score = (0.3110) × TMEM59L + (−0.0886) × CLCA1 + (0.1521) × TUBB2B was obtained. The patients were divided into a high-risk group and a low-risk group according to the median risk score for the Kaplan–Meier survival analysis. The results showed that the survival time of the high-risk patients was significantly lower than that of the low-risk patients (*p* < 0.001; Figure 6(b)). The AUC values of the 1-, 3-, and 5-year survival times predicted by the model were 0.600, 0.628, and 0.737 (Figure 6(c)). Risk score, survival time, and survival status were shown in the TCGA dataset, the risk scores from low to high, the survival time and survival status corresponding to the risk scores of different samples; and the gene expression in the prognostic model (Figure 6(d)). Furthermore, a multi-factor COX regression analysis of the risk score, tumor stage, age, and gender indicated that the risk score could be used as a prognostic factor independent of other clinical factors (Figure 6(e)).

### 3.7. TMEM59L, CLCA1, and TUBB2B Predicting Lymph Node Metastasis of CRC

**Figure 7:**
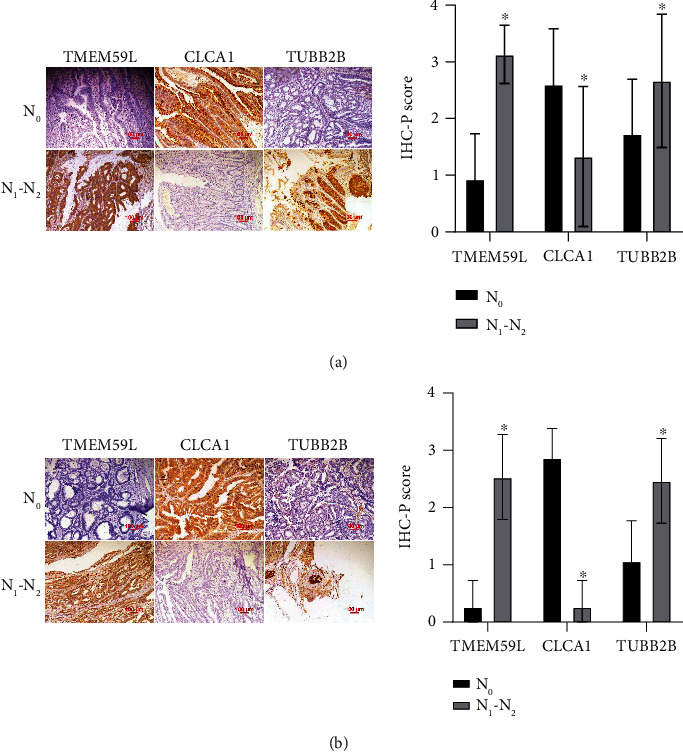
TMEM59L, CLCA1, and TUBB2B expression in colorectal cancer. (a) IHC staining of TMEM59L, CLCA1, and TUBB2B proteins in human rectal cancer tissues (200×) (N0: non-lymph node metastasis tissues; N1–N2: lymph node metastasis tissues). (b) IHC staining of TMEM59L, CLCA1, and TUBB2B proteins in human colon cancer tissues (200×) (N0: non-lymph node metastasis tissues; N1–N2: lymph node metastasis tissues).

In light of these observations and analysis in TCGA data, we hypothesized that TMEM59L, CLCA1, and TUBB2B may be the disease markers for lymph node metastasis of CRC. To test our hypothesis, we performed immunohistochemical staining on FFPE tissue sections from rectal cancer (Figure 7(a)) non-lymph node metastasis group (*n* = 15), lymph node metastasis group (*n* = 15), and colon cancer (Figure 7(b)) non-lymph node metastasis group (*n* = 15), lymph node metastasis group (*n* = 15). CLCA1 protein expression was lower in the lymph node metastasis group, while the TMEM59L and TUBB2B were higher in the lymph node metastasis group. These results were all statistically significant. Notably, these expression trends were consistent in both colon and rectal cancer.

## 4. Discussion

The lymph node metastasis of CRC is highly correlated with postoperative cancer recurrence and survival time [27]. Therefore, it is of great significance to find biomarkers that can predict early CRC lymph node metastasis. In this study, a total of 62 DEGs associated with CRC lymph node metastasis were discovered, of which 4 genes were down-regulated and 58 genes were up-regulated. A PPI network was constructed based on these DEGs, and the 20 hub genes were screened, among which only CLCA1 expression was down-regulated in patients with CRC lymph node metastasis, and the rest were up-regulated. Nine characteristic genes associated with lymph node metastasis were screened from the 20 hub genes using LASSO regression: KIF1A, TMEM59L, CLCA1, COL9A3, GDF5, TUBB2B, STMN2, FOXN1, and SCN5A.

KIF1A encodes a microtubule-dependent motor protein, which is responsible for the rapid anterograde transport of synaptic vesicle precursors in neurons [28]. KIF1A expression is significantly increased in ovarian cancer tissues. High KIF1A expression predicts poor prognosis. KIF1A may play a crucial role in biological processes, including positive regulation of T cell proliferation, primary immunodeficiency, pathways in cancer, the Wnt signaling pathway, and immune infiltrating cells [29]. Similarly, our research in CRC found that KIF1A is positively correlated with macrophages M0, B cells naive, and T cells CD4 memory activated. These findings suggest a potential role of KIF1A in modulating immune cell infiltration across different cancer types. TMEM59 is a recently discovered brain-specific high-expression protein that produces the effect of promoting apoptosis; nevertheless, the specific mechanisms of its apoptosis are still unclear [30]. TMEM59 deletion promotes the infiltration of inflammatory cells, macrophages, microglia cells, and neutrophils into the olfactory epithelium and lamina propria [31]. While downregulation of TMEM59 promoted anti-inflammatory factor expression and attenuated lipopolysaccharide treatment-induced inflammation [32]. CLCA1 is the first member of the CLCA family to be studied. This channel regulator family participates in a variety of cellular and molecular signal transduction pathways and plays a role in regulating cell proliferation, tumor invasion, and metastasis potential. It was discovered through study that its expression is decreased in the tumor tissues and serum of CRC patients [33], the OS rate of patients with low CLCA1 expression was significantly lower than that of patients with high CLCA1 expression, and the recurrence rate in patients with low CLCA1 expression was higher than that of patients with high CLCA1 expression [34]. COL9A3 encodes the collagen alpha-3 (IX) chain, which is a structural component of the hyaline cartilage and vitreous body of the eye [35]. In our study, we found that in CRC, COL9A3 is positively correlated with naive B cells and M0 macrophages, and negatively correlated with CD8 T cells and activated NK cells. This is somewhat parallel to the findings of Liu et al. [36] in esophageal squamous cell carcinoma, where the risk score, including COL9A3, was positively correlated with M1 and M2 macrophages. These findings suggest that COL9A3 may play a complex role in the tumor microenvironment across different types of cancer, influencing various immune cells' behavior. GDF5 encodes growth differentiation factor 5, which is a growth factor involved in bone and cartilage formation and regulates the differentiation of cartilage-forming tissues during cartilage development [37]. The absence of GDF5 may have consequences for immune responses and macrophage function in general and for arthritis in particular [38]. In our study, we found a positive correlation between KIF1A and macrophages M0 in CRC. TUBB2B encodes a tubulin beta-2b chain, where tubulin is the main component of the microtubules and plays a role in the correct axonal guidance of the central and peripheral axon bundles [39]. TUBB2B regulated the expression of TNF-a, IL-6, and PD-1/PD-L1 through the inhibit tumor invasion gene PER1 [40]. STMN2 encodes a microtubule stability regulator, which can regulate the assembly and decomposition of tubulin, stabilize microtubules, and thus control the lengths of the neurites in cortical neurons [41]. FOXN1 is a transcription regulator that regulates the development differentiation and function of thymic epithelial cells in the thymus [42]. FOXN1 mutation-mediated immune deficiency is typically associated with severe combined immunodeficiency [43]. FOXN1 has emerged as fundamental for thymus development, function, and homeostasis, representing the master regulator of thymic epithelial and T cell development [44]. We found that FOXN1 exhibited a positive correlation with T cells CD4 memory resting and a negative correlation with T cells follicular helper and T cells CD8. SCN5A encodes sodium channel protein 1.5 subtype *α* subunits, which are the major sodium channels in the heart tissue *α* subunits and form the main channels for sodium ion flow [45]. In this study, prognosis analysis showed that TMEM59L, CLCA1, and TUBB2B were survival-related genes in CRC, of which CLCA1 was a positive survival-related gene and TMEM59L and TUBB2B were negative survival-related genes.

Based on the KEGG analysis results provided, significant roles are suggested for the “ECM–receptor interaction,” “Circadian entrainment,” and “Protein digestion and absorption” pathways in lymph node metastasis of CRC. The interaction between the ECM and its receptors plays a pivotal role in various biological processes, including cell adhesion, migration, and signal transduction. Alterations in the ECM can influence the invasiveness and metastasis of tumor cells [46]. Circadian entrainment, or the regulation of biological clocks, has also been implicated in tumor development and progression [47]. Furthermore, the basic physiological process of protein digestion and absorption, which may change cancer, can impact tumor growth and development [48]. Recent research has identified a correlation between the expression of the GABRD gene in CRC and patient prognosis, suggesting potential involvement with pathways such as protein digestion and absorption, and ECM-receptor interaction [49]. These findings lend further support to our KEGG analysis results, although the precise mechanisms warrant further experimental validation.

Many studies have found that immune cells play an important role in tumorigenesis and the development of CRC, and tumor cells escape and inhibit the human immune system through a variety of means [50]. The lymph nodes are a kind of lymphoid tissue found throughout the body that can make immune cells resident and conduct immune surveillance on human tissues. Meanwhile, lymph node metastasis is also a common part of tumors. Cancer cells in lymph nodes can form their interactions with the host immune system by controlling the infiltration of the immune cells, suggesting that lymph node metastasis may be related to the infiltration of the immune cells [51]. This study indicated that the nine characteristic genes associated with lymph node metastasis were significantly correlated with immune cell infiltration, suggesting that tumor cells in CRC patients regulate immune cell infiltration through the characteristic genes of lymph node metastasis to form lymph node metastasis.

In this study, nine lymph node metastasis genes were analyzed via uni-factor COX regression, and three survival-related genes (TMEM59L, CLCA1, and TUBB2B) were ultimately identified. The risk score constructed from the expression levels of these three genes is an independent prognostic factor for CRC, suggesting that TMEM59L, CLCA1, and TUBB2B are closely related to the prognosis of CRC lymph node metastasis. We have verified this by immunohistochemical experiments in patient tissues. Up-regulation of CLCA1 and down-regulation of TMEM59L and TUBB2B may be effective methods of preventing the progression of CRC and prolonging the survival time of patients.

## 5. Conclusion

CRC tumor cells may regulate tumor immune cell infiltration through lymph node metastasis-related characteristic genes (KIF1A, TMEM59L, CLCA1, COL9A3, GDF5, TUBB2B, STMN2, FOXN1, and SCN5A) to form lymph node metastasis. Three survival-related genes (TMEM59L, CLCA1, and TUBB2B) were associated with the lymph node metastasis and survival time of patients.

## Data Availability

The data used to support the findings of this study are available from the corresponding author upon request.

## Abbreviations

AUC: Area under curve
CRC: Colorectal cancer
DEGs: Differentially expressed genes
DFS: Disease-free survival
FFPE: Formalin-fixed paraffin-embedded
GO: Gene Ontology
KEGG: Kyoto Encyclopedia of Genes and Genomes
LASSO: Least Absolute Shrinkage and Selection Operator
ROC: Receiver operating characteristic curve
TCGA: The cancer gene atlas
OS: Overall survival
PPI: Protein–protein interaction
ECM: Extracellular matrix.

## Supplementary Materials

We showed all the images to support the statistical results about immunohistochemistry in the four pdf files:

Supplementary Materials 1 rectal cancer N0.pdf

Supplementary Materials 2 rectal cancer N1-N2.pdf

Supplementary Materials 3 colon cancer N0.pdf

Supplementary Materials 4 colon cancer N1–N2.pdf.
