# Supplementary figures and images for: An Analysis of the Gene Expression Associated with Lymph Node Metastasis in Colorectal Cancer
Source: Int J Genomics. 2023 Sep 7;2023:9942663. doi: 10.1155/2023/9942663 (PMC10501847; doi:10.1155/2023/9942663)

Rectal Cancer  
N0

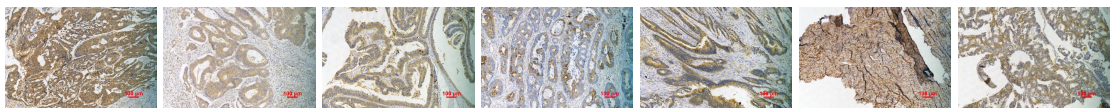

CLCA1

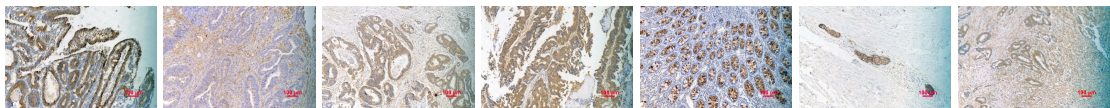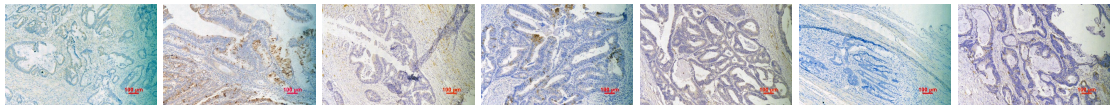

TUBB2B

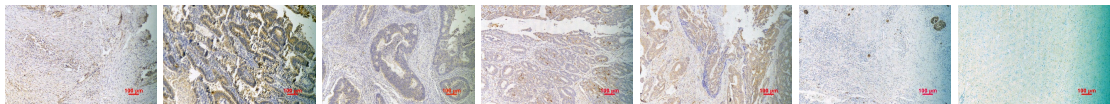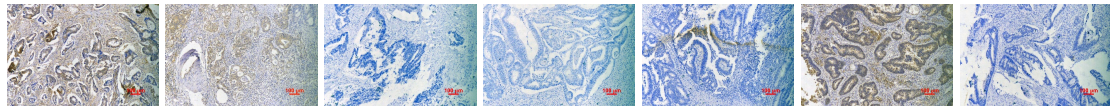

TMEM59L

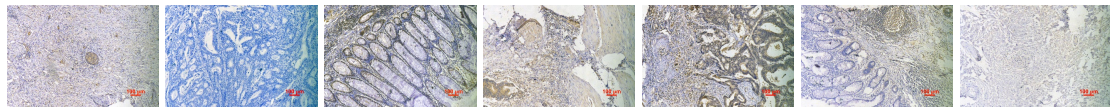

Supplement: Supplementary Materials — 1 rectal cancer N0.pdf [file 9942663.f1.pdf]

Rectal Cancer  
N1-N2

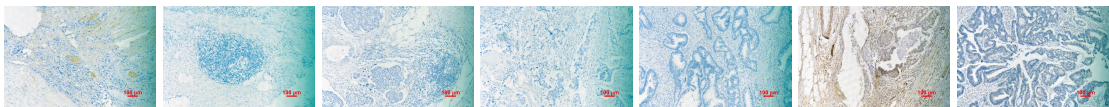

CLCA1

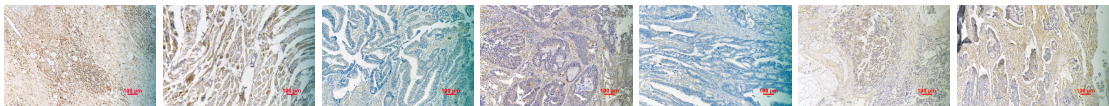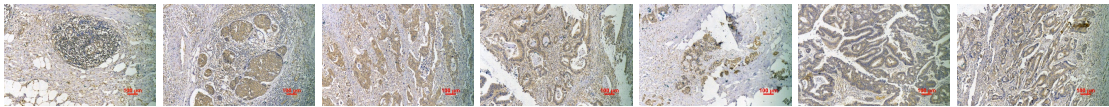

TUBB2B

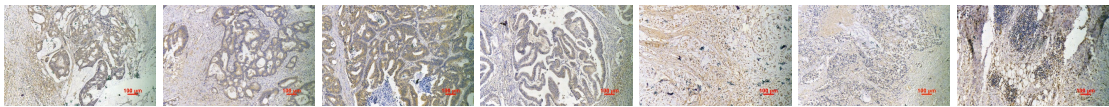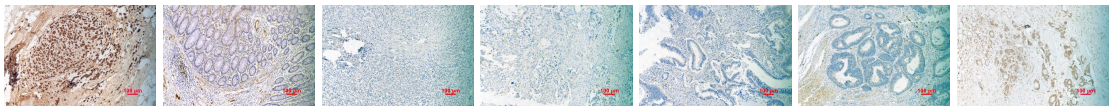

TMEM59L

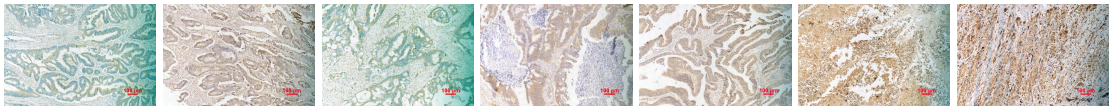

Supplement: Supplementary Materials — 2 rectal cancer N1-N2.pdf [file 9942663.f2.pdf]

Colon Cancer  
N0

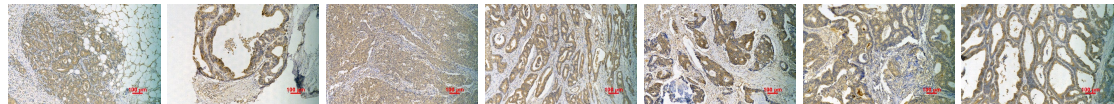

CLCA1

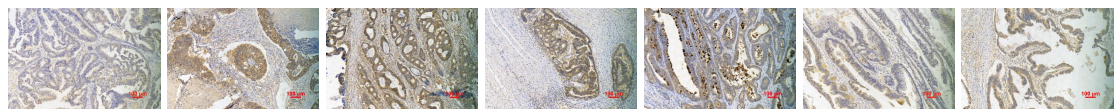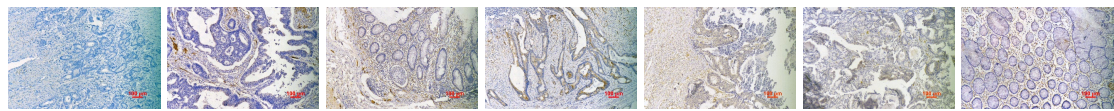

TUBB2B

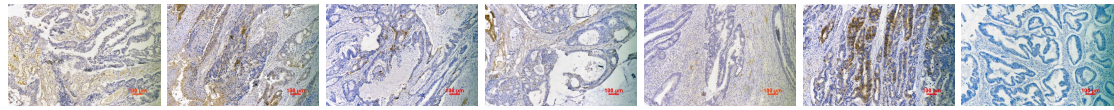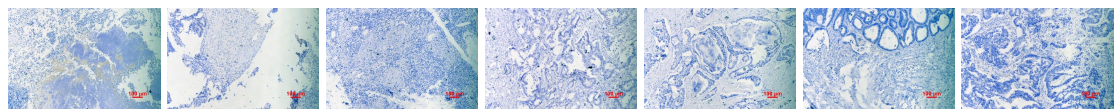

TMEM59L

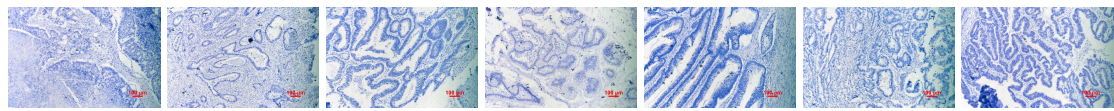

Supplement: Supplementary Materials — 3 colon cancer N0.pdf [file 9942663.f3.pdf]

Colon Cancer  
N1-N2

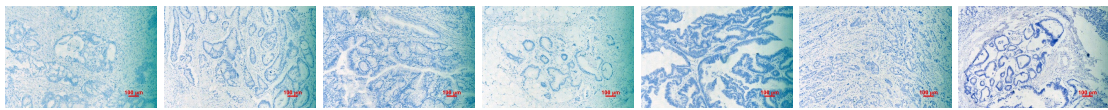

CLCA1

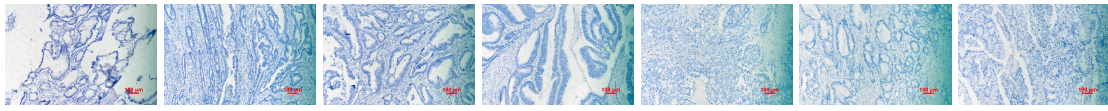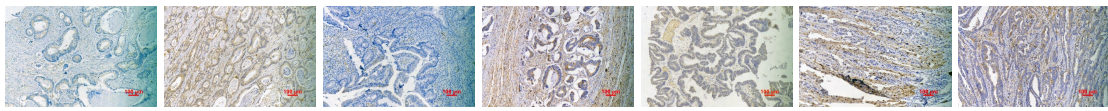

TUBB2B

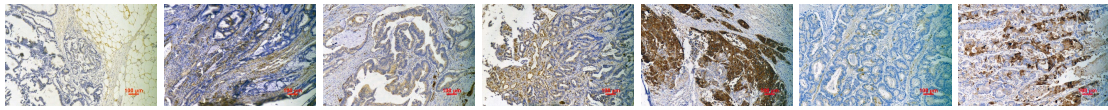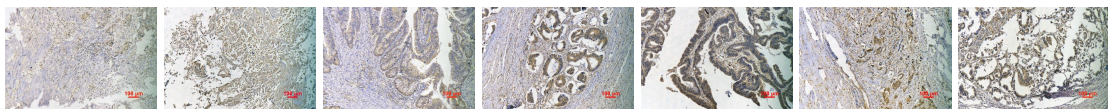

TMEM59L

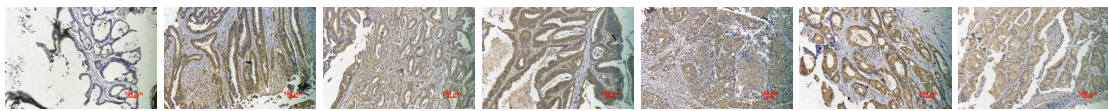

Supplement: Supplementary Materials — 4 colon cancer N1–N2.pdf. [file 9942663.f4.pdf]
